# Bilateral thalamic and mesencephalic infarctions with hypopituitarism as long-term complications postradiotherapy: A case report

**DOI:** 10.1097/MD.0000000000011917

**Published:** 2018-08-24

**Authors:** Liang Hui, Hu Shijun, Liu Tao, Wen Guoqiang, Huang Shixiong

**Affiliations:** Department of Neurology, Hainan General Hospital, Haikou, China.

**Keywords:** hypopituitarism, nasopharyngeal carcinoma, radiotherapy, thalamic infarction

## Abstract

**Background:**

Radiation is widely used as the first-line treatment for nasopharyngeal carcinoma (NPC) and improves survival. Nevertheless, radiation also places the patients at risk of radiation-induced adverse effects, such as transient ischemic attack, ischemic stroke, hypopituitarism, and cranial nerve and temporal lobe dysfunction.

**Case report:**

A 54-year-old woman who had undergone radiation treatment for NPC 14 years earlier and had no cerebrovascular risk factors, visited our department 4 days after sudden onset of consciousness disturbance. Brain magnetic resonance imaging (MRI) revealed bilateral thalamic and left mesencephalic infarctions with empty sella. Meanwhile, MR angiography showed narrowing in the bilateral posterior cerebral artery. Furthermore, laboratory tests showed low total triiodothyronine (T3), thyroxine (T4), free T3, free T4, luteinizing hormone, estradiol, follicle-stimulating hormone, and serum natrium and normal thyroid-stimulating hormone, which indicated radiation-related hypopituitarism. Serologically, she had low hemoglobin, hematocrit, mean corpuscular volume, mean corpuscular hemoglobin, mean corpuscular hemoglobin concentration, ferritin, and serum iron levels and elevated transferrin, manifesting microcytic anemia. The treatment, including aspirin, atorvastatin, levothyroxine, prednisone, saline infusion, and chalybeate, promoted the patient's recovery.

**Conclusion:**

To our knowledge, this is the first report of bilateral thalamic and mesencephalic infarction together with hypopituitarism following radiotherapy for NPC.

## 1 Introduction

Nasopharyngeal carcinoma (NPC) is known to be a familiar tumor in Southern China and to be sensitive to radiation therapy.^[1]^ Hence, radiation therapy results in improved 5-year survival.^[2]^ However, radiation entails hazards.^[3,4]^ Exposure to radiation can result in accelerated atherosclerosis; both large extracranial arteries and intracranial arteries are reportedly damaged after radiotherapy, which may further lead to stenosis of the cranial arteries and increased risk of cerebral infarction or transient ischemic attack.^[5]^ Therefore, radiation-induced cerebral infarction often develops after radiation treatment.^[6]^ On the contrary, the hypothalamic-pituitary axis is an extraordinarily radiosensitive range of the central nervous system, and hypopituitarism is usually induced by radiotherapy.^[7]^

We present the case of a middle-aged woman who showed bilateral thalamic and mesencephalic infarction along with hypopituitarism 14 years postradiotherapy for the treatment of an NPC.

## 2 Case report

**Figure 1:**
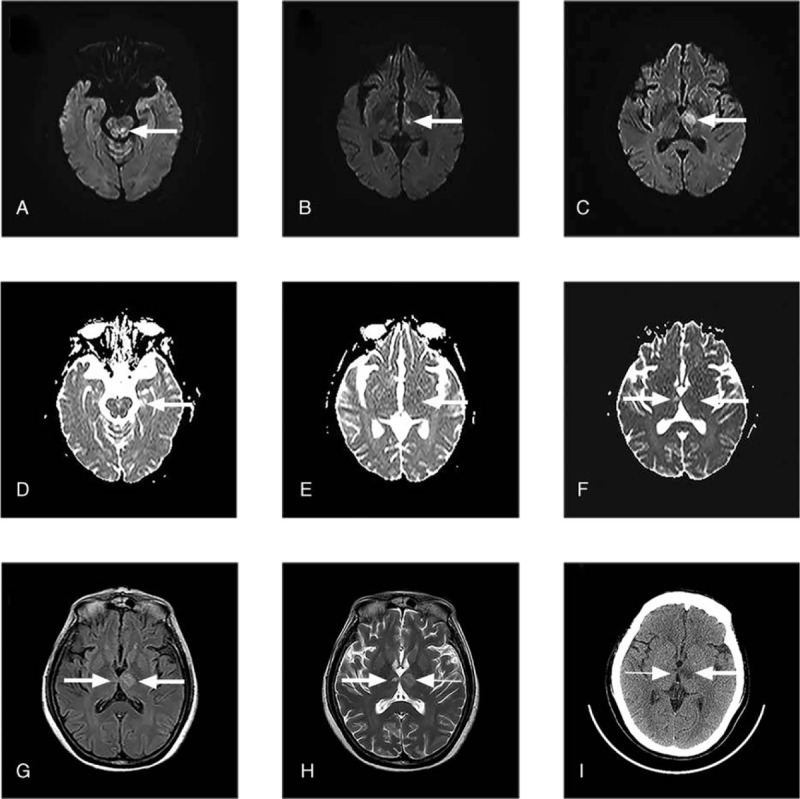
(A–C) Diffusion weighted axial images showing hyperintense signal in mesencephalic and left paramedian thalamic lesions (arrows). (D–F) Apparent diffusion coefficient images demonstrating decreased (or equal) signal in the same lesions and hyperintense signal in right paramedian thalamic lesion (arrows). (G, H) Axial FLAIR and T2 sequence images demonstrating hyperintense signal in the bilateral paramedian thalamic lesions (arrows). (I) Cerebral computed tomography showing bilateral paramedian thalamic lesions (arrows).

A 54-year-old woman was found unconscious by her family and was immediately transferred to the local hospital. Cranial computed tomography (CT) was performed several hours later and showed bilateral thalamic infarction (mainly left) (Fig. 1). The patient was treated with aspirin as an antiplatelet and atorvastatin to stabilize atherosclerotic plaque, and her consciousness gradually improved.

The patient was transferred to our department 4 days later. She had been diagnosed with an NPC 14 years earlier and had received radiation therapy (the specific radiologic dose and location are unknown). The cancer was successfully cured and had not relapsed. According to her daughter, her cranial CT was normal 2 years earlier and there had been no abnormalities in her behavior. Moreover, she had not been previously diagnosed with hypopituitarism. She neither smoked nor used medication and there was no history of hypertension, hyperlipemia, diabetes mellitus, atrial fibrillation, or cerebrovascular or coronary heart disease. Furthermore, her family history was unremarkable.

Her temperature (36.5°C), heart rate (70/min), blood pressure (115/73 mm Hg), and respiration (20/min) were all normal. Moreover, the respiratory, cardiovascular, and abdominal examinations were all within the normal limits. The neurologic examination showed hypersomnia, loss of memory, ptosis, and vertical movement disorder of the left eye. The pupils were isochoric, and the papillary light reflex was present. Her deep reflexes were normal; neither motor nor sensitive focal signs were detected and the Babinski sign was negative.

**Figure 2:**
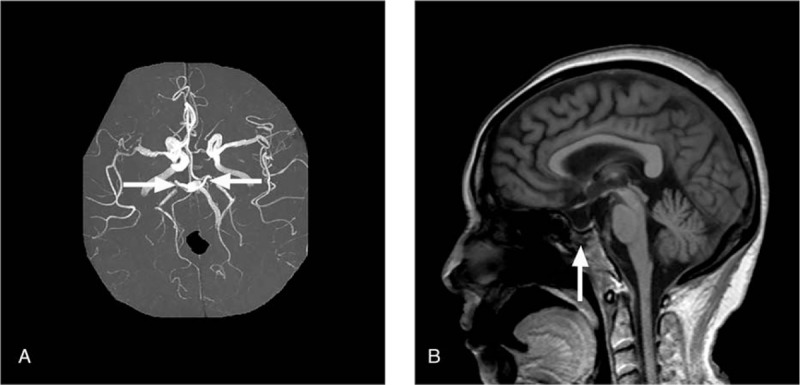
(A) Magnetic resonance angiography revealed whole brain cerebral atherosclerosis, especially bilateral posterior cerebral artery (arrows). (B) Sagittal T1-weighted image show the hypophysis has been injury severely (vacuole turcica) (arrow) and the nasal pharyngeal area is severely deformed.

Magnetic resonance imaging (MRI) was performed 2 days later and showed hyperintensities in the paramedian part of both thalami (the left was greater than the right) and left midbrain in fluid-attenuated inversion recovery and T2 sequences (Fig. 1). Additionally, there was hyperintensity of only the left paramedian thalamus and midbrain in the diffusion-weighted imaging (DWI) sequence and corresponding hypointensity based on the apparent diffusion coefficient (Fig. 1). The sagittal views revealed vacuole turcica and the nasal pharyngeal area was severely deformed. MR angiography (MRA) showed narrowing and irregular caliber of the whole brain cerebral artery, especially the bilateral posterior cerebral artery (Fig. 2). Unfortunately, due to noncooperation of the patient, further CT angiography or digital subtraction angiography was not accomplished. Written informed consent was obtained from the patient for the publication of this case report and any accompanying images.

The laboratory test results were as follows. Complete blood count showed lowered red blood cells, Hb, hematocrit, mean corpuscular hemoglobin (MCH), MCH concentration, mean platelet volume, mean corpuscular volume, platelet distribution width, elevated red blood cell distribution width, lowered ferritin, serum iron levels, and elevated transferrin (Table 1). Routine urine, blood sugar, lipid, hepatic, and renal function tests, 24 hours electrocardiogram monitoring, lupus anticoagulant, antiphospholipid antibodies, antinuclear antibodies, homocysteine, human immunodeficiency virus, and syphilis test were all within the normal limits. To further survey the cause of stroke in this patient, a transesophageal echocardiogram was performed and no patent foramen ovale was observed.

**Table 1:**
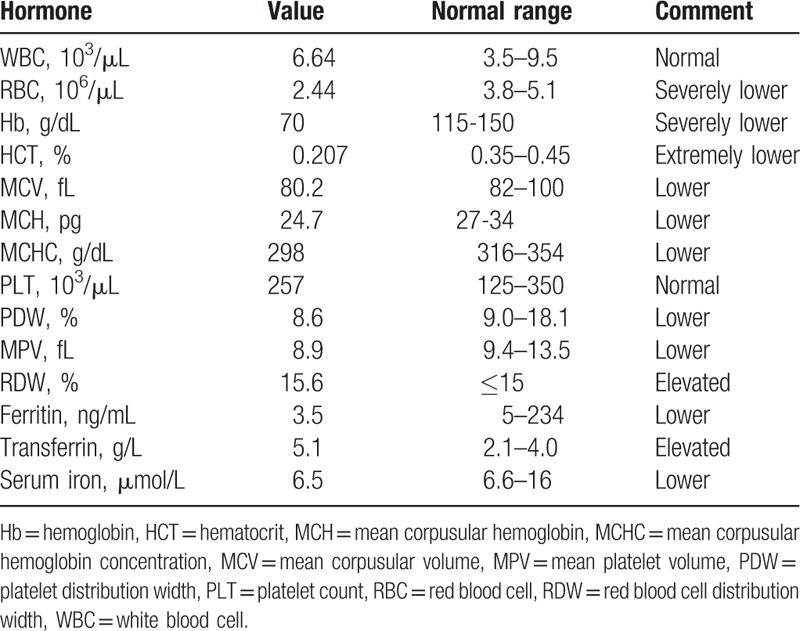
Complete blood count and anemia.

In addition, the laboratory data revealed lowered levels of total T3, free T3, total T4, free T4, luteinizing hormone (LH), follicle-stimulating hormone (FSH), estradiol, and cortisol accompanied by elevated prolactin, normal adrenocorticotropic hormone (ACTH), thyroid-stimulating hormone (TSH), and hyponatremia (Table 2). Secondary prevention of cerebral infarction including aspirin as anti-platelet aggregation, atorvastatin as stabilizing atherosclerotic plaque, and replacement therapy consisting of levothyroxine, cortisol, compound ferrous sulfate granules, and Ringer solution. About half a month later, the level of consciousness, ptosis, and movement disturbance of the left eye improved, but she remained amnesic. At the 1-year follow-up, her memory had not completely recovered but there was no recurrence of stroke.

**Table 2:**
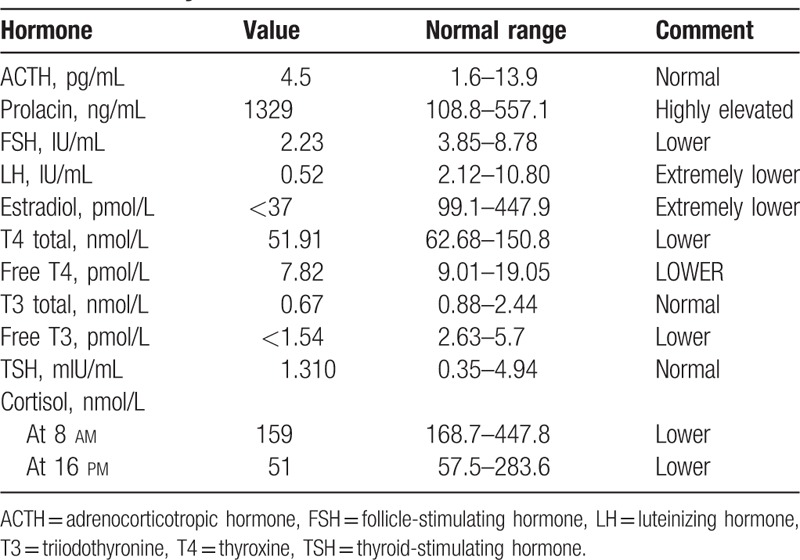
Endocrine analysis.

## 3 Discussion

To the best of our knowledge, most NPCs belong to poorly differentiated squamous cell carcinoma, which is sensitive to radiotherapy.^[8]^ However, successful treatment increases survival rate but places the patient at risk of radiation-induced adverse effects.^[9]^ Of these, irreversible chronic onset of hypopituitarism and vascular adverse effects are severe and could be life threatening.

The hypothalamic-pituitary axis lies directly within the radiation port; therefore, hypopituitarism is commonly induced by radiation therapy.^[7]^ Hypopituitarism is a progressive and irreversible chronic disorder, which can lead to damage of pituitary function, for example, dysfunction of the growth hormone axis, LH/FSH, and ACTH. On average, to lose 75% of the normal axis function of growth hormone may require 3.3 years, for LH/FSH, 7.8 years, for ACTH, 8.2 years.^[10]^ Moreover, hyperprolactinemia may also develop in postradiation hypopituitarism, perhaps deriving from deficient secretion of hypothalamic prolactin-inhibitory factor. The laboratory examinations combined with empty sella in the MRI of our patient were compatible with hypopituitarism. In addition, the hyponatremia in our patient is thought to be associated with secondary hypocortisolemia, which is characteristic of lacking adrenocorticotropin, which is responsible for inducing the adrenal gland to produce cortisol.^[11]^ Hence, it is advisable that pituitary and serum sodium evaluation should performed in patients who have received radiation to the hypothalamic-pituitary region, particularly in cases of neurologic dysfunction.

The primary sequelae of carotid atherosclerosis comprise of transient ischemic attacks, amaurosis fugax and carotid rupture, carotid wall thickening, and ischemic strokes. Reportedly, an interval of 6 months up to 20 years may intervene between irradiation and the first occurrence of clinical symptoms.^[12]^ Several studies^[13]^ have indicated that radiation therapy of head and neck malignancies increases the risk of cerebral arterial thrombosis. Shichita et al^[14]^ showed that apart from aging, hypertension, diabetes, hypercholesterolemia, smoking, and coronary heart disease, radiotherapy is also an independent risk factor of atherosclerosis.

The exact pathogenesis of radiation-related vascular injury remains uncertain, but there may be several mechanisms involved including fibrosis of the intima-media layer, damage of endothelial cells, occlusion of the vasa vasorum, and development of atheromatous plaques, which further give rise to carotid atherosclerosis.^[15]^ It has been reported that injury to the vasa vasorum and consequent ischemic lesions of the arterial wall are morphologic traits differentiating radiation-related arterial injury from spontaneous atherosclerosis. The arterial lesions caused by radioactive injury present with long and segmented damage.^[16]^ Some researchers^[14]^ have indicated that the most narrow position of carotid stenosis caused by radiation is located at the terminal of the lesion, while the most narrow location of carotid stenosis caused by factors other than radiation is confined to the middle of the lesion. The enlargement of plaque could accelerate blood-platelet aggregation, which promotes rapid development of carotid stenosis. Furthermore, the long and segmented damage of carotid stenosis could result in rapid carotid-artery stenosis. Therefore, antiplatelet drugs and statins may improve the carotid damage associated with radiotherapy.^[17]^ Intima-media thickness of the common carotid artery is a marker of early changes in atherosclerosis, which could be measured by modern high-resolution ultrasound machines and has proved to be a powerful predictor of future cerebrovascular disease.^[5]^ Early changes in postradiation carotid injury are asymptomatic; hence, it is recommended to regularly monitor the intima-media thickness of the common carotid artery after irradiation and perform timely interventions to avoid the occurrence of radiogenic vascular events.

The primary and secondary prevention of ischemic stroke caused by radiotherapy remains to be studied. Whether antiplatelet drugs, anticoagulation, and hypertension and hyperlipemia control can decelerate the development of the disease remain unknown. Intracranial artery stent implantation and endarterectomy are widely used as treatments for intracranial stenosis related to radiotherapy. However, Shin et al^[18]^ found that the proportion of stenosis after carotid angioplasty and stent implantation is much higher than in cerebrovascular disease of other etiology. Consequently, the optimal treatment for ischemic stroke caused by radiotherapy remains to be discovered.

The thalamus is a large ovoid complex that consists of several nuclei with diverse functions and is symmetrically distributed on either side of the ventriculus tertius. The thalamus is a subcortical sensory conduction receiving and relaying station, which serves as the pathway of communication between the cerebral cortex and midbrain; it contains 5 major functional nuclei: the reticular and intralaminar nuclei in charge of arousal and nociception, effector nuclei participating in motor function and aspects of language, limbic nuclei involved with mood and motivation, associative nuclei concerned with high-level cognitive functions, and sensory nuclei in all major regions.^[19]^ Vascular lesions differentially damage these nuclei and result in behavioral and sensorimotor syndromes depending on which nuclei are affected. More specifically, the reticular and intralaminar nuclei are involved in regulating consciousness, alertness, and sleep; consequently, thalamic infarction especially in the paramedian part may lead to increase in sleep. Serious somnolence or coma may develop if the structure is destroyed. Goyal et al^[20]^ reported the case of a woman with severe somnolence whose neurologic examination and electroencephalogram were normal, eventually confirmed as bilateral thalamic collateral injury in MRI after excluding pharmacologic, toxic, and metabolic factors. Other studies^[21]^ have indicated apparent increasing demand in sleep after thalamic infarction, particularly bilateral thalamic infarction. These studies support the notion that the thalamus plays an important role in controlling sleep activity.

**Figure 3:**
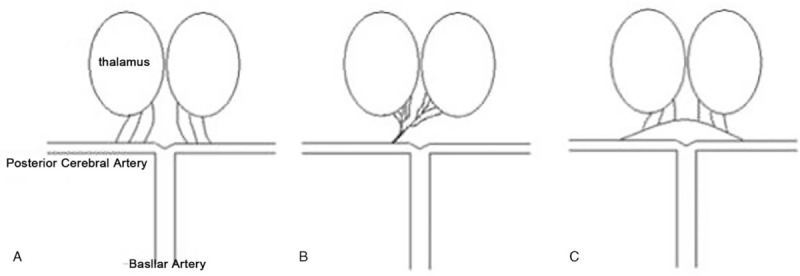
Three types of paramedian thalamic-mesencephalic arterial supply described by Percheron. (A) Type I: Most common variant, where a perforating artery arises from each P1 segment. (B) Type II: The artery of Percheron arises from one P1 segment and splits to supply the bilateral thalamic and rostral midbrain. (C) Type III: An arcade of perforating arteries arising from an artery bridging the bilateral P1 segments.

Generally speaking, the thalamus is supplied both by the anterior and posterior circulations, mainly involving 4 arteries: the tuberothalamic artery, thalamic perforating artery, thalamic genicular artery, and posterior choroidal artery.^[20]^ The anterior thalamus is supplied by the tuberothalamic artery deriving from the posterior communicating artery through the anterior circulation. The paramedian thalamus and rostral midbrain are supplied by the thalamic perforating artery, which is usually branched by the P1 segment of the posterior cerebral arteries. To the best of our knowledge, there are 3 types of variation, as described by Percheron.^[22]^ Type I, which is the most common, is described as bilateral perforating arteries originating from each P1 segment. Type III involves an arcade of perforating arteries originating from the artery bridging the P1 segments of both PCAs. Type II is rare; a single perforating artery (which is also called Percheron artery [PA]) arises from one P1 segment and separates to supply the bilateral thalami and rostral midbrain (Fig. 3).^[19]^ PA occlusion results in bilateral paramedian thalamic infarcts, with or without involvement of the midbrain. The typical triad of acute PA occlusion is: alteration of consciousness, loss of memory, and vertical movement disorder of the eye.^[23]^ The clinical manifestation of the patient in this study resembled the typical triad, combined with bilateral thalamic paramedian lesions in CT. First, we assumed that the patient had PA occlusion, yet our assumption was not verified by the subsequent MRI, revealing different stage infarction of the bilateral thalami. According to the cranial MRI and the lack of stroke history, the patient had presented with silent cerebral infarction of the right thalamus within the past 2 years. The responsible congested vessel was attributed to a right thalamic perforating artery. Why the patient had no abnormal clinical manifestations after infarction of the right thalamus occurred remains unclear. Maybe it is because the first right thalamic infarction involved the right thalamic reticular and intralaminar nuclei, yet the brainstem reticular structure could still project to the cerebral cortex through the left thalamic reticular and intralaminar nuclei, protecting the patient from symptoms when the first infarction occurred. Nevertheless, the occlusion involved the left thalamic perforating artery during the second incident, leading to the damage of the left thalamic reticular and intralaminar nuclei, further resulting in the altered level of consciousness. Loss of memory is due to damage in associative nuclei of the left thalamus. Ptosis and vertical gaze palsy were considered to be related to the mesencephalic lesions, possibly connected to the third cranial nerve and rostral midbrain tegmentum, containing the interstitial nucleus of Cajal.

Typical bilateral parathalamic infarction is infrequent, with sudden unexpected somnolence or coma usually being the common clinical manifestation. However, it is easy to neglect, which is ascribed to lack of examination of memory and eye movement. Our report aims at emphasizing that clinicians should consider bilateral thalamic infarction after excluding other diseases such as toxicosis, infection, hypoglycemia, electrolyte disturbance, diabetic hyperosmotic coma when encountering sudden consciousness alterations. Except from PA occlusion, acute unilateral thalamic infarction could lead to coma, loss of memory, and oculomotor disorder when the other side of the thalamus had already been injured, especially when occlusion of a thalamic perforating artery is involved. Therefore, we should perform MRI and DWI as soon as possible when encountering patients with disorder of consciousness.

Our patient showed cerebral atherosclerosis, especially narrowing of the bilateral posterior cerebral artery on MRA and thalamic and mesencephalic infarction on MRI. It is unfortunate that the patient refused cerebral angiography to further confirm the cerebral atherosclerosis. Apart from radiotherapy, the patient had neither risk factors of atherothrombotic brain infarction nor relative family and medication history. On the contrary, the patient developed hypopituitarism after receiving radiotherapy, and we speculate that the cerebral infarction was related to the radiotherapy.

In the meantime, the patient was diagnosed with microcytic anemia as evidenced by hypothyroidism (Table 2), low Hb, mean corpusular volume, and ferritin (Table 1). The pituitary gland carried weight on erythropoiesis, and anemia was supposed to be related with lack of thyrotrophic and adrenotropic hormones.^[24]^ The low ferritin level hinted at poor iron absorption by the duodenal lumen.^[25]^ In such cases, replacement therapy with thyroid hormone, cortisol saline infusion, and chalybeate are effective strategies.

All in all, to increase tumor-related survival rate, regular assessments of carotid artery intima-media thickness and anterior-pituitary function are necessary in such patients to accomplish a timely diagnosis and enable a suitable therapy.

## 4 Conclusion

Damage caused by radiotherapy is a chronic, progressive, and irreversible process. In this patient, 14 years passed from the radiotherapy until the development of cerebral infarction and hypopituitarism complicated with second-stage hypothyroidism, hypocortisolemia, hyponatremia, and microcytic anemia. Secondary prevention of cerebral infarction and replacement therapy benefited her life quality.

Bilateral thalamic and mesencephalic infarctions usually indicate PA occlusion, but clinicians should not neglect acute unilateral thalamic and mesencephalic infarction in cases where infarction has occurred in the contralateral thalamus.

To our knowledge, we are the first to report such rare bilateral thalamic and mesencephalic infarctions along with hypopituitarism in a single patient.

## Author contributions

**Conceptualization:** Wen Guoqiang.

**Supervision:** Liu Tao, Huang Shixiong, Hu Shijun.

**Writing - original draft:** Liang Hui.

**Writing - review & editing:** Liang Hui, Huang Shixiong, Hu Shijun.
